# Cytotoxic T-lymphocyte antigen-4 +49A/G polymorphisms in Sudanese adults with type 1 diabetes and latent autoimmune diabetes

**DOI:** 10.1186/s13104-019-4814-y

**Published:** 2019-11-26

**Authors:** Shimos A. Alshareef, Saeed M. Omar, Hamdan Z. Hamdan, Ishag Adam

**Affiliations:** 1 Al-Ghad International College for Applied Medical Sciences, Medina, Kingdom of Saudi Arabia; 2 Central Laboratory, Khartoum, Sudan; 3 Faculty of Medicine, Gadarif University, Gadarif, Sudan; 4 Faculty of Medicine, Al-Neelain University, Khartoum, Sudan; 5 Faculty of Medicine, University of Khartoum, P.O. Box 102 Khartoum, Sudan

**Keywords:** Autoantibodies, Cytotoxic T-lymphocyte antigen-4, Latent autoimmune diabetes in adults, Polymorphism, Type 1 diabetes mellitus, Sudan

## Abstract

**Objectives:**

This study was conducted to assess the association of T-lymphocyte-associated protein 4 (*CTLA-4* +49A/G) variant with Latent autoimmune diabetes in adults (LADA) in Eastern Sudan. The study included 24 LADA, 240 patients with type 1 diabetes mellitus (T1DM), and 240 healthy controls. Genotyping for *CTLA*-*4* +49A/G was done by polymerase chain reaction restriction fragment length polymorphism (PCR-RFLP).

**Results:**

Genotypes distribution of *CTLA-4* in controls was in accordance with the HWE (*P *> *0*.05). The frequency of mutation (both homozygous and heterozygous) of *CTLA*-*4* +49A/G (AG + GG) was significantly higher in LADA compared with T1DM and the controls [19 (79.1%) vs. 100 (41.7%) vs. 78 (32.5%), P < 0.001]. It was significantly higher when LADA was compared with T1DM [19 (79.1%) vs. 100 (41.7%), P = 0.018, OR = 3.21, 95% CI 1.16–8.89] and when LADA was compared with the controls [19 (79.1%) vs. 78 (32.5%), P = 0.001, OR = 4.49, 95% CI 1.62–12.42]. The rate of heterozygous mutation of the CTLA-4 +49A/G (AG) was significantly higher in LADA compared with T1DM and the controls [16 (66.7%) vs. 85 (35.4%) vs. 70 (29.2%), P < 0.001]. It was significantly higher when LADA was compared with T1DM [16 (66.7%) vs. 85 (35.4%), P = 0.002, OR = 3.64, 95% CI 1.49–8.87] and when LADA was compared with the controls [16 (66.6%) vs. 85 (35.4%), P = 0.001, OR = 4.85, 95% CI 1.98–11.86].

## Introduction

Latent autoimmune diabetes in adults (LADA) is the term coined to type 1 diabetes mellitus (T1DM) which has similar clinical features of type 2 diabetes (T2DM) mellitus, however it has slower immunizing damage of β-cells than found in T1DM [1]. LADA accounts for 4–12% of adults with T2DM [2, 3].

The detection of circulatory islet cell autoantibodies [glutamic acid decarboxylase antibodies (GADAs) and islet antigen-2 (IA-2As)] which are the main features for T1DM and LADA, could explain the beta cell damage which is caused by cytotoxic T lymphocytes [4]. Although, the genetic basis of LADA is not yet fully understood, previous studies have reported that LADA patients have shared some genetic features with both T1DM and T2DM [5, 6]. Cytotoxic T-lymphocyte antigen-4 (*CTLA*-*4*) can act a co-stimulatory molecule which is expressed on activated T lymphocytes and possess a key role in autoimmunity via down regulating T-cell function [7]. The CTLA-4 gene is located on chromosome 2 (2q33) and it has been shown that CTLA-4 is associated with various autoimmune diseases such as T1DM, Graves’ disease and asthma [7–10].

There are few published researches on association between LADA and *CTLA*-*4* [5, 11–14] and there is no existing publication on association between LADA and *CTLA*-*4* in Sub-Saharan Africa including Sudan. Diabetes is a big health problem in Sudan [15]. The current study was conducted to investigate the role of *CTLA4* on the susceptibility of LADA in eastern Sudan.

## Main text

### Methods

A case–control study was conducted in Gadarif Teaching Hospital clinic in the eastern Sudan during the period of February through August 2017. The cases (were part of the patient who had the initial clinical diagnosis of T2DM) were 24 patients with LADA which was diagnosed by the criteria of their age at onset of diabetes was > 30 years, did not require insulin for at least 6 months after diagnosis and the presence of circulating GAD65 antibodies [4]. Two groups of controls were selected. The first group of controls were 240 patients who presented with T1DM to the hospital during the study period and who developed diabetic ketosis or ketoacidosis at onset and required insulin treatment at the time of diabetes diagnosis. The other control group were 240 healthy individuals (age‐ and sex‐matched) with no family history of diabetes or autoimmune diseases. A total of 240 (44.5 ± 3.6 years; M/F:118/122) clinically characterized T2DM patients, who had been on oral treatment for at least 3 months, were screened for LADA by detecting the presence of GAD65 and IA-2 autoantibodies in the serum using ELISA as the manufacturer described (Anti-GAD/IA2 pool ELISA Test, Euroimmune, Germany). From these T2DM group, 24 patients were identified and labelled as LADA cases.

**Fig. 1:**
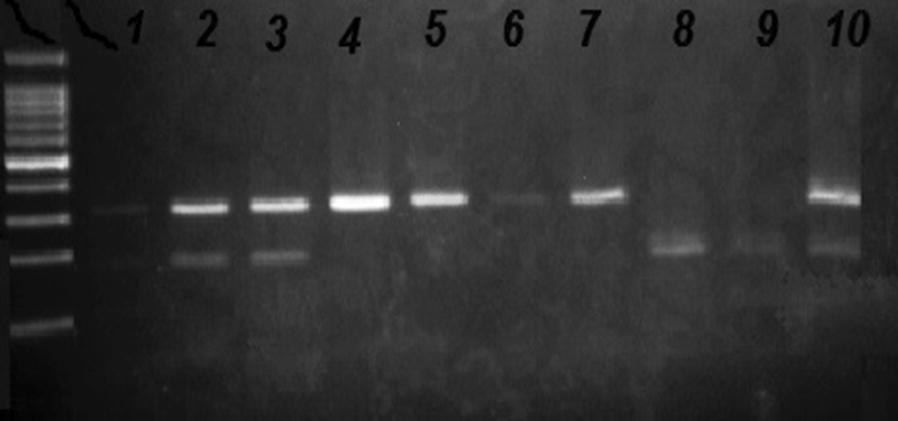
PCR-RFLP analysis for *CTLA*-*4* +*49A/G* polymorphism. Two fragments of 74/88 bp indicate homozygous (G/G), three fragments of 74, 88 and 162 bp indicate heterozygous genotype (A/G) and undigested fragment of 162 bp indicate homozygous A/A genotype. Lane L show the 50 bp ladder; Lane1 blank control

The LADA positive samples were selected for genotyping analysis for *CTLA*-*4* +*49A/G* gene polymorphism, were compared to a similar number of T1DM patients and normal control subjects. PCR-restriction fragment length polymorphism (PCR-RFLP) in +49A/G position was used for genotyping. Genomic DNA from peripheral blood leukocytes was extracted as described previously [16]. PCR was performed using Forward primer (5′-GCTCTACTTCCTGAAGACCT-3′) and Reverse primer (5′-AGTCTCACTCACCTTTGCAG-3′) according to the previous method [17]. PCR products were subjected to restriction digestion with BbV1 restriction enzyme (Fermentas Life Sciences, Germany) using the supplier’s protocol. The restriction products were proceeded in 2% agarose gel electrophoresis in order to visualize the *CTLA-4* sequence (162-bp). If a G was found at position 49, *BbvI* cut the sequence, resulting in 88/74-bp fragments, while the sequence was intact if an A was located in the same position, Fig. 1.

### Statistics

SPSS was used for data analyses. The clinical data were compared between LADA, T1DM and the controls by *t*-*test* and *Chi square* test for continuous and categorized data, respectively. Hardy–Weinberg equilibrium (HWE) was performed to compare the observed frequencies of the different genotype distribution with their expected frequency in the controls groups by using Pearson’s Chi square (χ^2^) statistical test [18]. The genotypes and alleles difference between the LADA, T1DM and the controls were compared by Pearson’s Chi square (χ^2^) statistical test. A two-sided *P*-value < 0.05 was considered statistically significant.

Genotype risk was calculated by multiplying the genotype of the SNP for each individual by the effect size (β-coefficient) and summing the individual score as described previously [19]. Binary regression analysis was used to build risk model by using phenotype LADA as a dependent factor and the covariates e.g. age, sex and body mass index (BMI) as independent factor. Firstly, covariates age, sex and BMI were calculated in model 1. Then weighted genetic risk score was entered as a single covariate in model 2. At last all covariates including weighted genetic risk score were included in model 3. Both, *R*^*2*^ and *X*^*2*^ were calculated for each model.

### Results

The three groups were matched in their age and sex. LADA antibodies were observed in 24 patients (10.0%). Healthy controls showed no autoantibodies.

Genotypes distribution of *CTLA-4* in controls was in accordance with the HWE (*P *> *0*.05).

The frequency of mutation (both homozygous and heterozygous) of *CTLA*-*4* +49A/G (AG + GG) was significantly higher in LADA compared with T1DM and the controls [19 (79.1%) vs. 100 (41.7%) vs. 78 (32.5%), P < 0.001]. It was significantly higher when LADA was compared with T1DM [19 (79.1%) vs. 100 (41.7%), P = 0.018, OR = 3.21, 95% CI 1.16–8.89] and when LADA was compared with the controls [19 (79.1%) vs. 78 (32.5), P = 0.001, OR = 4.49, 95% CI 1.62–12.42]. There was no significant difference in the *CTLA*-*4* +49A/G mutation between the T1DM and the controls, Table 1.

**Table 1:** The frequency (%) of *CTLA*-*4* +*49 A/G* polymorphism

Genotype frequencies	LADA (24)	T1DM control (240)	Healthy control (240)	P	OR	95% CI
AA	5 (20.8)	140 (58.3)	162 (67.5)	All < 0.001		
				LADA vs. T1DM = 0.001	0.18	0.06–0.52
				LADA vs. controls = 0.001	0.12	0.04–0.35
				T1DM vs. controls = 0.037	0.67	0.46–0.97
AG	16 (66.7)	85 (35.4)	70 (29.2)	All < 0.001		
				LADA vs. T1DM = 0.002	3.64	1.49–8.87
				LADA vs. controls = 0.001	4.85	1.98–11.86
				T1DM vs. controls = 0.143	1.33	0.90–1.95
GG	3 (12.5)	15 (6.3)	8 (3.3)	All 0.087		
				LADA vs. T1DM = 0.598	0.74	0.20–2.50
				LADA vs. controls = 0.598	0.74	0.20–2.50
				T1DM vs. controls = 0.990	1.00	0.61–1.61
AG + GG	19 (79.1)	100 (41.7)	78 (32.5)	All < 0.001		
				LADA vs. T1DM = 0.018	3.21	1.16–8.89
				LADA vs. controls = 0.001	4.49	1.62–12.42
				T1DM vs. controls = 0.067	1.73	0.97–2.00
Allele A	26 (54.2)	365 (76.0)	394 (82.1)	All < 0.001		
Allele G	22 (45.8)	115 (24.0)	86 (18.0)	LADA vs. T1DM < 0.001	0.37	0.20–0.68
				LADA vs. controls < 0.001	0.25	0.13–0.47
				T1DM vs. controls = 0.021	0.69	0.50–0.94

The rate of heterozygous mutation of the CTLA-4 +49A/G(AG) was significantly higher in LDA compared with T1DM and the controls [19 (79.1%) vs. 100 (41.7%) vs. 78 (32.5%), P < 0.001]. It was significantly higher when LADA was compared with T1DM [19 (79.1%) vs. 100 (41.7%), P = 0.018, OR = 3.21, 95% CI 1.16–8.89] and when LADA was compared with the controls [16 (66.6%) vs. 70 (14.6%), *P *= 0.001, OR = 4.49, 95% CI 1.62–12.42], Table 1. The homozygous rate of the CTLA-4 + 49A/G (GG) mutation was not different between the LADA, T1DM and the controls, Table 1.

Regression analysis showed that, weighted genetic risk score was a significant risk factor for developing LADA in model 3, OR = 2.82 (1.71–4.65); *P *= <0.001. Likewise, BMI remained as a risk factor in model 3 when it was combined with genetic risk score, OR = 1.03 (1.01–1.06); *P *= 0.003, Table 2.

**Table 2:** Regression model for evaluating genetic risk of LADA patients

Variables	OR (95% CI)	*P*-value
Model 1		
Age (years)	0.99 (0.94–1.05)	0.901
Sex	1.42 (0.62–3.26)	0.404
BMI, kg/m^2^	1.03 (1.00–1.05)	0.013
*X*^*2*^= 6.3761		0.095
*R*^*2*^= 0.04		
Model 2		
wGRS	2.53 (1.56–4.11)	**< ***0.001*
*X*^*2*^=13.84		< 0.001
*R*^*2*^= 0.085		
Model 3		
wGRS	2.82 (1.71–4.65)	**< ***0.001*
Age (years)	0.99 (0.94–1.05)	0.863
Sex	1.35 (0.57–3.19)	0.480
BMI, kg/m^2^	1.03 (1.01–1.06)	*0.003*
*X*^*2*^ = 22.6		< 0.001
*R*^*2*^= 0.138		

*BMI* body mass index, *CI* confidence intervals, *OR* odds ratio, *wGRS* weighted genetic risk score

### Discussion

The current study showed a higher frequency of heterozygous mutation of *CTLA*-*4* +*49A/G* in the LADA compared with T1DM and the controls. However, the rate of the homozygous mutation of *CTLA*-*4* +*49A/G* was not different between the LADA, T1DM and the controls. It seems to be this is the first report on association of *CTLA*-*4* +*49A/G* genotypes and diabetes mellitus in Sub-Saharan Africa. Previous studies reported a significant association between the *CTLA*-*4* +*49 A/G* polymorphism and the susceptibility to LADA and T1DM in different settings e.g. China [11, 13], Estonia [20, 21], Spain and Italy [22], Poland [23] and Argentina [14]. Furthermore, recent systematic review demonstrated that *CTLA*-*4* +*49A/G* polymorphism is associated with LADA [9].

On the other hand Delitala et al.,-who have investigated *CTLA-4 G6230A* variant in Sardinian population (Italy)—reported no significant difference in the distribution of G6230A genotypes and alleles in LADA patients, early and late-onset T1D patients, and healthy controls [12]. Likewise Shih et al., observed no significant association between *CTLA*-*4* +*49* genotypes with T2DM [24]. It noteworthy that, *CTLA*-*4* +*49 A/G* is a missense variant that is located in exon 1 at position 49. This missense variant resulted in changing the hydrophilic amino acid threonine to a non-polar amino acid alanine [10]. The replaced amino acid is located in the leading sequence of the CTLA-4, which may affect the folding or migration of the CTLA-4 protein towards the T-cell receptor. From another prospective, Ueda et al., confirmed that, carrier of homozygous risky genotype G/G express about half of the amount that expressed by the subject who have a protective homozygous genotype A/A [7]. Moreover, Anjos and his colleagues reported that, CTLA-4 peptide translated from G/G genotype individuals is subjected to mal-handling of the peptide by endoplasmic reticulum. This is result in glycosylation of the CTLA-4 peptide which decrease the subsequence cellular expression. On the other hand individuals who have the protective genotype A/A have a well handled and processed CTLA-4 peptide and end up with successful cellular expression [25]. Perhaps, this provide the rational of malfunctioning and loss of the T-cell inhibition observed in activated cytotoxic T-cell functions in patients with autoimmune diseases [26].

It is worth to be mentioned that our results should be cautiously compared with the results of the later studies because of the ethnicity difference between the various settings.

### Conclusion

The *CTLA*-*4* +*49A/G* polymorphism is associated with development of LADA in this setting.

## Limitations of the study

In number (hence the sample size) of the LADA was small in this study and this might have effect of the power. Other markers and genotypes were not investigated. Gadarif is located on Ethiopian border and Sudanese population might not be of the same ethnicity.

## Data Availability

The datasets used and/or analysed during the current study are available from the corresponding author on reasonable request.

## Abbreviations

LADA: latent autoimmune diabetes in adults
CTLA4: cytotoxic T-lymphocyte associated protein 4
T1DM: type 1diabetes mellitus
T2DM: type 2 diabetes mellitus
